# Predictive Processing during a Naturalistic Statistical Learning Task in ASD

**DOI:** 10.1523/ENEURO.0069-19.2020

**Published:** 2020-12-10

**Authors:** Neelima Wagley, Renee Lajiness-O’Neill, Jessica S. F. Hay, Margaret Ugolini, Susan M. Bowyer, Ioulia Kovelman, Jonathan R. Brennan

**Affiliations:** 1 Department of Psychology and Human Development, Vanderbilt University, Nashville, TN 37205; 2 Department of Psychology, University of Michigan, Ann Arbor, MI 48109; 3 Department of Psychology, Eastern Michigan University, Ypsilanti, MI 48197; 4 Department of Psychology, University of Tennessee, Knoxville, TN 37996; 5 Department of Neurology, Henry Ford Hospital, Detroit, MI 48202; 6 Department of Linguistics, University of Michigan, Ann Arbor, MI 48109

**Keywords:** ASD, development, language, MEG, statistical learning

## Abstract

Children’s sensitivity to regularities within the linguistic stream, such as the likelihood that syllables co-occur, is foundational to speech segmentation and language acquisition. Yet, little is known about the neurocognitive mechanisms underlying speech segmentation in typical development and in neurodevelopmental disorders that impact language acquisition such as autism spectrum disorder (ASD). Here, we investigate the neural signals of statistical learning in 15 human participants (children ages 8–12) with a clinical diagnosis of ASD and 14 age-matched and gender-matched typically developing peers. We tracked the evoked neural responses to syllable sequences in a naturalistic statistical learning corpus using magnetoencephalography (MEG) in the left primary auditory cortex, posterior superior temporal gyrus (pSTG), and inferior frontal gyrus (IFG), across three repetitions of the passage. In typically developing children, we observed a neural index of learning in all three regions of interest (ROIs), measured by the change in evoked response amplitude as a function of syllable surprisal across passage repetitions. As surprisal increased, the amplitude of the neural response increased; this sensitivity emerged after repeated exposure to the corpus. Children with ASD did not show this pattern of learning in all three regions. We discuss two possible hypotheses related to children’s sensitivity to bottom-up sensory deficits and difficulty with top-down incremental processing.

## Significance Statement

Language acquisition involves segmenting the continuous speech stream into sounds, syllables, and words. Learning these units relies on both the properties of the input, as well as emerging high-order cognitive mechanisms that guide learning from the top-down. We examined the neurobiology underlying the integration of top-down and bottom-up information in statistical speech segmentation in children with and without autism spectrum disorder (ASD). We offer evidence of neural and behavioral effects of syllable-to-syllable processing in speech segmentation that differ in typically developing children from children with a clinical diagnosis of ASD. Our findings inform developmental and cognitive theories of language acquisition by examining the computational nature of speech segmentation across different populations of learners.

## Introduction

Language acquisition involves segmenting continuous speech into sounds, syllables, and words. By detecting statistical regularities in the input, learners can incrementally anticipate upcoming information for subsequent word learning. For instance, after 2 min of exposure to a foreign language, infants begin to identify statistically frequent syllable sequences and treat those as labels for novel objects (Hay et al., 2011). Learning the linguistic units relies on the properties of the input; it is a bottom-up driven cognitive process. In parallel, experience and high-order cognitive mechanisms also guide this learning process from the top-down (Kuhl, 2004; Werker, 2018). However, little is known about the neurobiology underlying the integration of bottom-up and top-down information in statistical speech segmentation. This is an important knowledge gap that impedes our understanding of acquisition in typical development and neurodevelopmental disorders that impact language acquisition, such as autism spectrum disorder (ASD; Tager-Flusberg et al., 2005). We investigate neural signals underlying statistical learning in children with and without ASD using magnetoencephalography (MEG).

Behavioral work suggests that children with ASD may be as equally equipped as their neurotypically developing (NT) peers to use statistical patterns to find words in speech (Obeid et al., 2016). For example, Mayo and Eigsti (2012) varied the likelihood that syllables co-occur [transitional probability (TP)] in a 21 min long corpus and found similar segmentation outcomes for children with and without ASD. Scott-van Zeeland et al. (2010) also found comparable learning performance between NT and ASD children after exposure to a continuous speech stream. Importantly, the groups differed in their neural responses. With increased exposure to the input, NT children showed reduced activation in a fronto-temporal-parietal network, while children with ASD did not show task related changes in brain activity.

Both prior studies used artificial language materials which lacked varying prosodic and stress patterns integral to everyday speech (Johnson and Jusczyk, 2001), thus, leaving open the question of how individuals would perform given more natural language input. Indeed, children with ASD may struggle to find words in natural speech for at least two reasons. MEG studies show that children with ASD have a delayed mismatch response to speech and non-speech sounds (Roberts et al., 2011) and demonstrate atypical responses to irregular speech sound sequences (Brennan et al., 2016b; Galilee, et al., 2017). This may indicate potential deficits in bottom-up early sensory processing of speech. We label this the sensory-differences hypothesis.

In addition, children with ASD have difficulty extracting global regularities (“weak central coherence”; Frith, 1989) and allocating attention within sound sequences (Whitehouse and Bishop, 2008), which may be a disadvantage in the types of top-down processing necessary for statistical learning. Such differences are supported by reduced patterns of activation in a network of fronto-temporal regions associated with typical language acquisition (Redcay and Courchesne, 2008) which are more pronounced in children who have poor language learning outcomes (Lombardo et al., 2015). We label this the prediction-differences hypothesis. We propose that early sensory deficits and/or atypical predictive processing may lead to difficulties in extracting statistical regularities from fluent speech.

We asked children to listen to naturally spoken passages in Italian with a range of TPs between syllables. We quantify TP using the information processing metric of surprisal, defined as the inverse-log of conditional probability between two syllables (for details, see Materials and Methods; Hale, 2016). We apply this metric for the first time to measure syllable-to-syllable prediction in natural speech with a focus on children with and without ASD. To tease apart the hypotheses, we track evoked neural responses for syllables in left hemisphere regions implicated in key steps of speech processing (Hickok and Poeppel, 2007): early perception in the primary auditory cortex (LAC), mapping percepts to linguistic units in the posterior superior temporal gyrus (pSTG), and higher-order analysis of linguistic regularities in the inferior frontal gyrus (IFG). Passages were repeated three times to capture a neural index of learning, defined as change in the evoked amplitude as a function of surprisal across repetitions. In NT children, we expect to see the index of learning across all three regions of interest (ROIs). As surprisal increases, amplitude of the evoked neural response should increase; this sensitivity should emerge after repeated exposure to the passages. Crucially, this effect may differ between the NT and ASD groups. The sensory deficit hypothesis holds that ASD individuals will show reduced sensitivity to surprisal in early sensory regions, such as the left LAC and pSTG. The prediction hypothesis holds that children will show reduced sensitivity to surprisal within higher order regions like the left IFG.

## Materials and Methods

### Participants

Fifteen children with ASD (1 female, M_age_ = 10.00, SD =* *1.16) and fourteen age and gender matched NT children (M_age_ = 10.06, SD =* *1.46) participated in the study. All children (age range = 8–12 years) were prescreened for eligibility through a phone interview with a parent or caregiver and were monolingual English speakers. The study was approved by all participating institutional review boards, as part of a larger project assessing language and communication in ASD using MEG (Brennan et al., 2016b; Lajiness‐O'Neill et al., 2018; Brennan et al., 2019). Parents and children provided informed consent and assent and received monetary compensation for their participation.

### Inclusion and exclusion criteria

Participants were recruited through local clinics and communities in southeast Michigan. During prescreening, caregivers completed the social communication questionnaire (SCQ; Rutter et al., 2003). The SCQ is a 40-item caregiver screening to assess communication and social functioning in individuals who may have an ASD. Items referenced across the symptomology domains of ASD are totaled for a single score and a cutoff classification score of 11 is often used for research purposes (Rutter et al., 2003). To participate in the current study, ASD-likely candidates required a SCQ ≥ 11 (Corsello et al., 2007), and NT participants required a SCQ < 11.

The behavior assessment system for children (BASC; Reynolds and Kamphaus, 2002) and the Wechsler abbreviated scale of intelligence-2 (WASI-2; Wechsler and Hsiao-pin, 2011) were administered to rule out adaptive and intellectual deficits consistent with intellectual disability. The BASC measures general behaviors and emotions of children such as hyperactivity, aggression, and conduct problems. The WASI-2 is a brief and reliable measure of intellectual functioning and includes subtests tapping into verbal, nonverbal, and general cognition. Inclusion criteria for all participants included at least low average intelligence [full-scale IQ (FSIQ) ≥ 80; Wechsler and Hsiao-pin, 2011].

A formal diagnosis of all ASD-likely participants was based on the *Diagnostic and Statistical Manual of Mental Disorders*, Fifth Edition (DSM-5; American Psychiatric Association, 2013) diagnostic criteria and the autism diagnostic observation schedule (ADOS), administered by a clinical and research reliable psychologist (Lord et al., 2012). The ADOS is a semi-structured standardized assessment of communication, play, social interaction, and restricted and repetitive behaviors. To confirm the diagnosis of ASD, the ADOS Module 3 was administered. The revised algorithm (see Gotham et al., 2007) was used to compute individual and a combined total score for subdomains of social interaction, communication, and stereotyped behaviors/circumscribed interests. Participants with ASD had a combined total score above the clinical cutoff suggestive for autism (Gotham et al., 2007).

Exclusionary criteria for ASD and NTs included any known history of head injury with loss of consciousness, other neurologic disorders including active epilepsy/seizures, environmental deprivation, anxiety disorders or other forms of psychopathology, and anything that might interfere with the MEG procedure (e.g., dental braces). Additional exclusion criteria for NTs included any history of developmental delay or a first-degree relative with an ASD diagnosis. Two NT participants were excluded from analyses because of equipment error during MEG data acquisition and one ASD participant was excluded because of an inability to comply with the task demands and tolerate the assessment procedures. The final group of 29 did not significantly differ in age or gender (see Table 1).

**Table 1:** Mean (standard deviations) of standardized assessments

		NT		ASD			
	*N*	Mean (SD)	*N*	Mean (SD)	*t*	*p*	*g*
Gender (M:F)		13:1		14:1			
Age (years)		10.00 (1.64)		10.06 (1.47)	–0.10		
CTOPP phonological awareness (standard score)	14	91.00 (13.36)	15	94.07 (18.99)	−0.51	0.617	–0.18
TOPS inferences	14	103.86 (8.57)	11	86.00 (20.07)	2.86	0.012	1.15
TOPS predicting	14	104.07 (11.85)	13	80.54 (17.25)	4.10	<0.001	1.55
CELF formulating sentences (scaled score)	10	14.60 (1.26)	7	8.86 (5.27)	2.83	0.028	1.56
CELF concepts and following directions	13	10.69 (2.29)	13	7.62 (4.77)	2.10	0.051	0.77
NEPSY auditory attention	14	11.14 (1.96)	15	7.47 (4.66)	2.80	0.011	0.98
WASI FSIQ (*t* score)	13	114.62 (8.17)	13	97.54 (19.23)	2.95	0.009	1.11
BASC (standard score)	14	44.29 (5.47)	15	61.8 (4.57)	−9.32	<0.001	−3.38
SCQ (total score)	14	1.43 (1.95)	15	18.60 (7.53)	−8.53	<0.001	−2.98
ADOS total			15	7.90 (2.85)			

*t* statistic and *p* values are reported for a two-tailed independent samples test. Effect sizes are reported using Hedges’ *g.*

FSIQ, full-scale IQ measure from the Wechsler abbreviated scale of intelligence-2; CTOPP, comprehensive test of phonological processing; TOPS, test of problem solving; CELF, clinical evaluation of language fundamentals; NEPSY, developmental neuropsychological assessment; BASC, behavior assessment system for children; SCQ, social communication questionnaire.

### Experimental design

**Figure 1.**
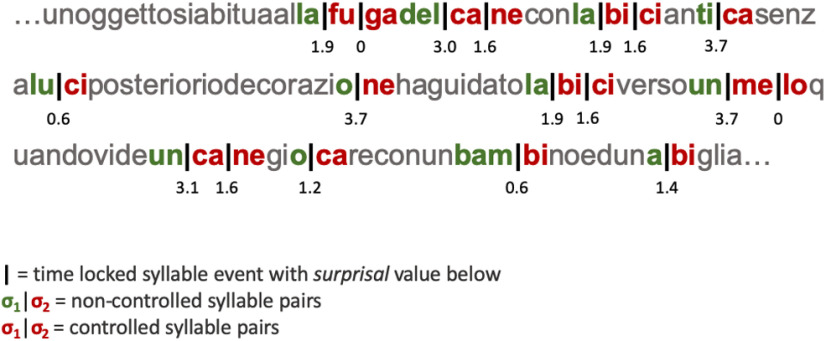
Schematic of the experimental stimuli as adapted from Hay et al. (2011). An excerpt of the ∼2-min-long Italian passage showing key target (controlled) syllables (red) and non-controlled syllables (green) pairs. The passage was repeated three times for a total duration of ∼6 min.

Participants passively listened to ∼6 min of a naturally produced passage in a foreign language (Italian) modeled after stimuli previously used by Hay et al. (2011; see Fig. 1). The Italian passage consisted of grammatically plausible but semantically nonsensical sentences made up of legal Italian words. To ensure natural production of Italian pronunciations, a female native Italian speaker recorded three different instances of the passage. Each participant listened to all three versions (three repetitions) presented via E-Prime Software 2.0 (Schneider et al., 2002). Of interest were the relative distributions and occurrences of eight key target syllables (*fu*, *ga*, *me*, *lo*, *ca*, *ne*, *bi*, *ci*) presented throughout the passage. A trigger signal marking the onset of each passage segment was used to pinpoint the time signature of these syllables, which was then aligned with the continuous MEG signal.

**Figure 2.**
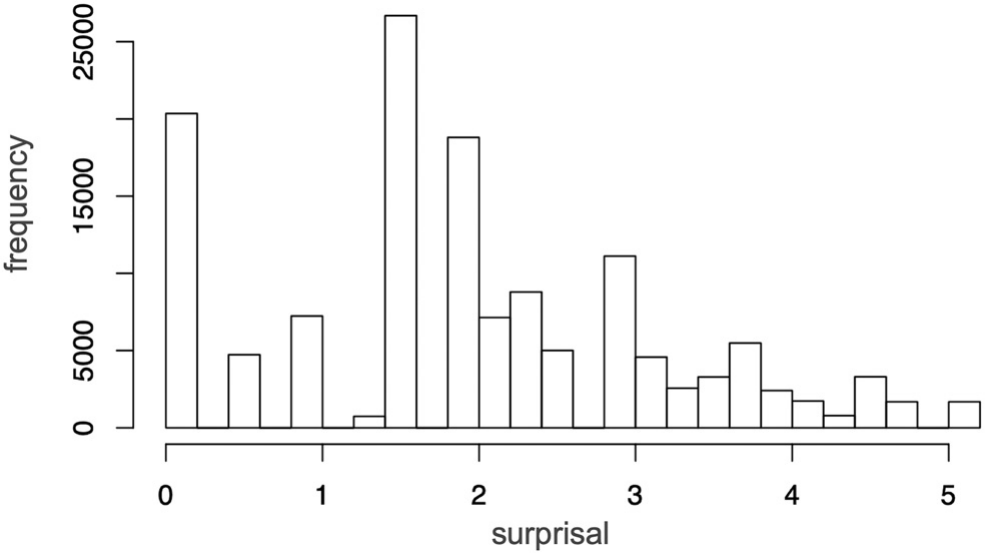
Histogram of the range of surprisal distributions of surprisal values across all target syllables.

We tracked the exact timing of the syllable occurrences and the resulting brain responses given the following methodological manipulation. For each occurrence of a target syllable, the forward internal TP between its preceding syllable and the target syllable was calculated [i.e., frequency of target syllable given frequency of preceding syllable; TP = P(σ_2_|σ_1_)]. TP for all target syllables ranged from 0.028 to 1.00. These TP values were converted to surprisal [surprisal = -log_2_(TP)] as prior work on phonological and lexical processing has shown that linguistic frequencies affect processing on a logarithmic scale (Hale, 2001, 2016). This yielded a total of 576 surprisal values for each presentation of the target syllables across the three repetitions of the passage for each participant (for distributions of surprisal, see Fig. 2). This metric of surprisal allows us to measure, in a continuous way, the information conveyed by a linguistic event, such as the likelihood of a particular syllable, based on its given context. Thus, a context of low syllable-to-syllable TP yields high surprisal and high syllable-to-syllable TP yields low surprisal.

The surprisal metric taps into the brain’s sensitivity to statistical regularities at multiple levels of representation (Hale, 2001; Levy, 2008). Prior work with surprisal has documented behavioral and neurobiological measures on adults at syntactic (Monsalve et al., 2012; Frank et al., 2013; Gwilliams and Marantz, 2015; Willems et al., 2016; Brennan et al., 2016a; Lopopolo et al., 2017; Gwilliams et al., 2018) and lexical or phonemic levels of processing (Gwilliams and Marantz, 2015; Lopopolo et al., 2017; Gwilliams et al., 2018).

The target syllables were drawn from four target words (*fuga*, *melo*, *cane*, *bici*) that were systematically placed throughout the corpus. The component syllables of *fuga* and *melo* (*fu*, *ga*, *me*, *lo*) appeared nowhere else in the passage, giving these words a high TP = 1.0 (surprisal = 0.0). In contrast, the component syllables of *cane* and *bici (ca*, *ne*, *bi*, *ci*) appeared within the passage another 24 times each (e.g., *taCI*, *CAro*), thus giving them a lower TP = 0.33 (surprisal = 1.585). The syllables of these words appeared 36 times within the passage, only 12 of which were in the target words and the others as initial (e.g., *CAdi*), medial (e.g., *sindaCAto*), or final (e.g., *spreCA*) syllables. The inclusion of these four legal Italian words, comprised of the key target syllables, allowed us to control and test for statistical learning effects of relatively moderate and highly predictive syllable sequences within a continuous and varied range of syllable probabilities.

### Behavioral measures

After listening to the Italian passages, a statistical learning post-test was given outside the scanner to explicitly measure children’s ability to distinguish words with TP of 1.0 and 0.33 from novel Italian words that did not occur within the corpus. Children listened to a pair of words presented via E-Prime Software 2.0. One of the two words was a bi-syllabic target word from the passages and the other word (non-target) was one of four bi-syllabic Italian words comprised of syllable combinations that were not included in the Italian corpus (e.g., *mugo*, *azza*, *pipa*, *zebu*). However, the component syllables of these novel words did appear in the Italian passages (e.g., *mu*). Children were tested using a two-alternative forced-choice task by asking, “Which of the following two words could be a possible word in the language you just heard?” Participants were instructed to press the “1” key if the first word could be a possible word in the foreign language, and similarly, to press the “2” key if the second word could be a possible word in the foreign language. Children completed four practice trials using common English words (e.g., *teacher*) versus nonsense, phonotactically illegal words (e.g., *pmfkin*) followed by 16 trials of the Italian target and non-target pairs of words.

Standardized measures of language and attention were also obtained as part of the larger project investigating language and communication in children with ASD (see Table 1). For the purpose of this particular study, we simply report descriptive statistics on a subset of these measures to note the language and communication skills of the ASD group studied here, and for discussion in relation to the previous studies of statistical learning in children with ASD (Scott-van Zeeland et al., 2010; Mayo and Eigsti, 2012). Measures of language include the comprehensive test of phonological processing (CTOPP; Wagner et al., 1999), clinical evaluation of language fundamentals (CELF-5; Semel et al., 2006), and test of problem solving (TOPS 3; Bowers et al., 2005) to assess phonological, syntactical, grammatical, and pragmatic competence, respectively. A test of auditory attention included the Auditory Attention subtests of the NEPSY developmental neuropsychological assessment (NEPSY-II; Korkman et al., 2007).

### Procedure

Participants completed the neuroimaging portion (∼10 min), immediately followed by the behavioral statistical learning test, and lastly, the behavioral battery of language and cognitive assessments (60–90 min). Participants laid supine on a bed with a helmet-shaped dewar containing 148 magnetometer MEG sensors placed around their head (4D Neuroimaging). Children were instructed to keep their eyes open (monitored via video) and listen to the foreign language while remaining as still as possible. During scanning, the stimuli were delivered via computer speakers placed at an aperture in the shielded room; loudness was set at a comfortable level for each participant.

### Data acquisition and processing

Three small electrode coils, used to transmit head location information to the neuromagnetometer probe, was affixed to each participant’s forehead with two-sided tape. Additional localization coils were attached to each preauricular point (PA), anterior to the tragus of the ear on the two sides of the head. Standard automatic probe position routines (4D Neuroimaging Hardware) were used to locate the five coils placed on the head with respect to the neuromagnetometer detector coils and to digitize the shape of the head for co-registration to a standard MRI. Neuromagnetic fields were recorded with a whole-head 148-chanel magnetometer (WH 2400, 4D Neuroimaging system). During acquisition, the data were bandpass filtered between 0.1 and 100 Hz and digitally sampled at 508.63 Hz. Data were recorded continuously for later analyses. The onset of each repetition of the Italian passage was recorded as pulse codes whose strength indicated the type of stimulus on a trigger channel collected simultaneously with the MEG data. The location of events on the trigger and response channels were used to select epochs from −0.3 to 1 s of MEG data around each target syllable for each 2-min repetition of the passage. Data analysis was performed using the Fieldtrip toolbox for EEG/MEG-analysis (Oostenveld et al., 2010).

Extracranial sources of interference were attenuated by subtracting signals recorded by five gradiometer and six magnetometer reference channels placed ∼15–20 cm from the head. Epochs were filtered using a discrete Fourier transform filter at 60, 120, and 180 Hz with a 2-s padding and a high pass filter at 0.5 to attenuate line noise. Trials and channels containing artifacts were removed based on visual inspection. No >23 channels of 148 and 106 trials of 576 were removed during artifact rejection (mean trials removed ASD = 50, NT = 58). The two groups did not significantly differ on the total number of channels (*t*_(27)_ = 0.11, *p *=* *0.74) or trials (*t*_(27)_ = 2.07, *p *=* *0.16) removed.

### ROIs analysis

Source time courses were reconstructed on to a 7- to 11-year-old pediatric template brain (Fonov et al., 2011) at four ROIs using Montreal Neurologic Institute (MNI) coordinates. Three ROIs were selected a priori based on previously reported findings on statistical learning paradigms in the speech domain with adults (Karuza et al., 2013), which included left primary auditory cortex (*x* = −48, *y* = 18, *z* = 2), posterior region of the left STG (*x* = −64, *y* = −12, *z* = 4), and left IFG (BA 44; *x* = −52, *y* = 26, *z* = −6). We also included a right superior parietal region (*x* = 24, *y* = −46, *z* = 60) as a control ROI.

Single-trial source-localized time courses were estimated using a linear constrained minimum variance (LCMV) beamformer (Van Veen et al., 1997). The LCMV beamformer forms a linear combination of the external field measurements to monitor the activity at a single brain location, while optimally suppressing all other noise and other source contributions to the MEG data. The beamformer filter was estimated using a sensor covariance matrix based on the average of all epochs per participant. MEG sensor averages were then projected through the filter for each location, yielding source time courses in three dimensions for each ROIs. The root-mean-square (RMS) time course within three 100-ms time bins (Teinonen et al., 2009): 200–300, 250–350, and 300–400 ms following syllable onset, at each location, per participant, per trial, for each repetition of the passage was entered into the statistical analysis. Time windows of interest were chosen based on two related accounts: first, prior work shows consistent modulation of the evoked response between 200 and 500 ms during statistical segmentation of a syllable stream (Sanders et al., 2002; Cunillera et al., 2006); second, theoretical frameworks of speech perception suggest that temporal sampling of the speech stream for syllables occurs over longer intervals, roughly 150–300 ms, and that this time window carries syllable-boundary and syllabic-rate cues as well as other prosodic and stress cues relevant for the type of perceptual processing assessed here (Näätänen and Picton, 1987; Poeppel, 2003; Hickok and Poeppel, 2007; Giraud and Poeppel, 2012).

### Statistical analysis

To test for a neural index of learning, we measured the relative change in evoked response amplitude as a function of surprisal across the repeated passages. A linear mixed-effects model was fit using the lmer function in the lme4 package in R (Bates et al., 2015) with passage repetition, ROI, group, and time window as categorical variables and surprisal as a continuous variable (all as fixed effects). Variation among participants was taken into account by including individuals as a random effect intercept; *p* values were computed via the Satterthwaite approximation using the lmerTest package in R. Statistical inference was based on *F* tests of main effects and higher order interactions using the anova function in R. We excluded 54 trials from statistical analyses corresponding to target syllables with only one occurrence (i.e., a trivial case of TP = 1.0, surprisal = 0).

Additionally, a Bayesian multilevel model was fit using the brms package (Bürkner, 2017) with the same parameters as mentioned above. Models were fit using two chains of 1000 warm-up iterations and 2000 sampling iterations. Prior distributions on all terms were the default values from brm(). To report on the key manipulations of interest (e.g., change in evoked response as a function of surprisal for third repetition between NT and ASD groups), we extracted the mean β coefficient and the 95% credible interval (CI) for the slope of the amplitude over surprisal as sampled from the posterior distribution of the model. All model terms had a R-hat value ≤1.01.

For behavioral responses on the statistical learning task, the proportion of correct responses was calculated out of 16 trials from 14 NT and a subset of 12 ASD participants who completed the task (three ASD children did not complete the postscan behavioral test because of computer error and/or inability to comply with the task demands).

### Code accessibility

The brms model output described in the paper is freely available online at Open Science Framework, https://osf.io/zbvhc/.

## Results

### Statistical learning behavioral results

**Figure 3.**
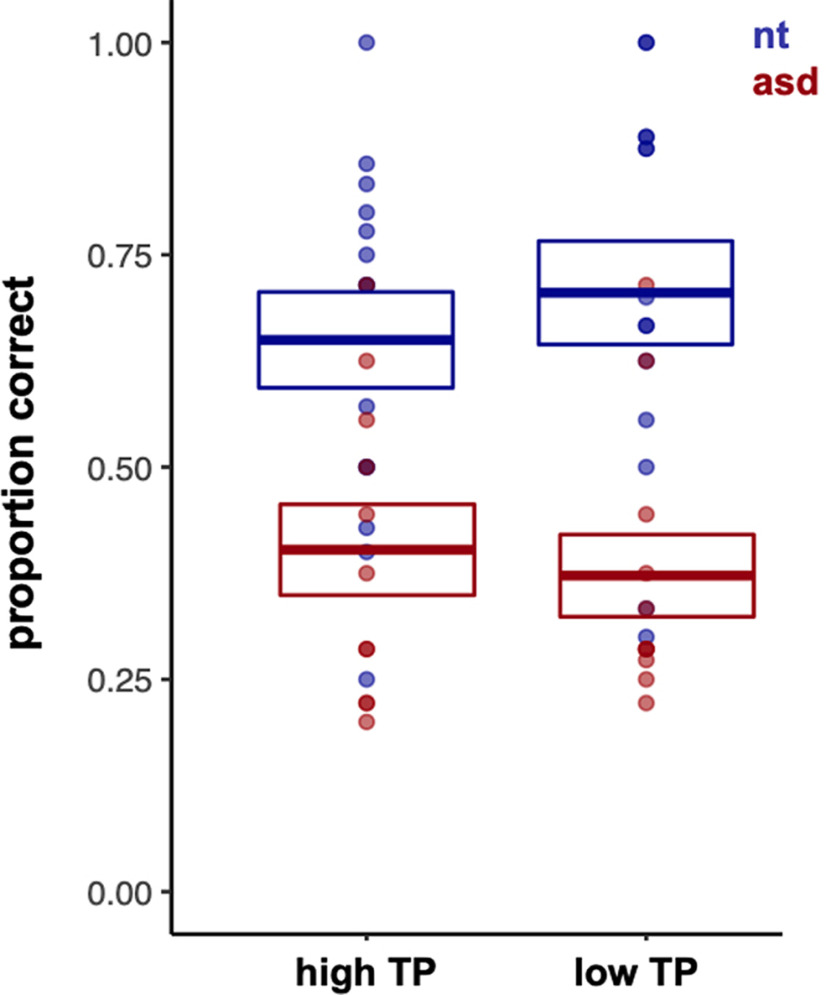
Proportion of correct responses to high and low TP target words in comparison to novel Italian words, calculated out of 16 trials from 14 NT and a subset of 12 ASD children who completed the behavioral learning test. Error bars represent standard error.

Performance on the Italian behavioral test is shown in Figure 3. A two-way ANOVA [group (NT, ASD) × TP (high, low)] revealed there was a significant main effect of group (*F*_(1,48)_ = 24.3, *p *<* *0.001, η_p_^2^ = 0.34). NT children outperformed children with ASD in correctly identifying both the high TP (*t*_(24)_ = 2.78*, p *=* *0.002, Cohen’s *d *=* *0.97) and low TP (*t*_(23)_ = 4.33, *p *=* *0.001, *d *=* *1.28) words from novel Italian words, as revealed by independent sample *t* tests. There was no group by TP interaction effect (*F*_(1,48)_ = 0.95, *p *=* *0.33, η_p_^2^ = 0.02). In both groups, there were no differences in accurately identifying high TP from low TP words in comparison to novel Italian words (no main effect of condition; *F*_(1,48)_ = 0.02, *p *=* *0.89, η_p_^2^ = 0.00). Therefore, accuracy on all trials were averaged as one and counted as total proportion of correct responses for each group and tested against chance (i.e., 0.5). One-sample *t* tests showed that NT children had above-chance accuracy in identifying the target-words [M (SD) = 0.68 (0.17); *t*_(13)_ = 3.93, *p *=* *0.002, *d *=* *0.13], whereas children with ASD performed below chance in accurately identifying the target words [M (SD) = 0.40 (0.14), *t*_(11)_ = −2.71, *p *=* *0.02, *d* = −1.23].

### MEG results

**Figure 4.**
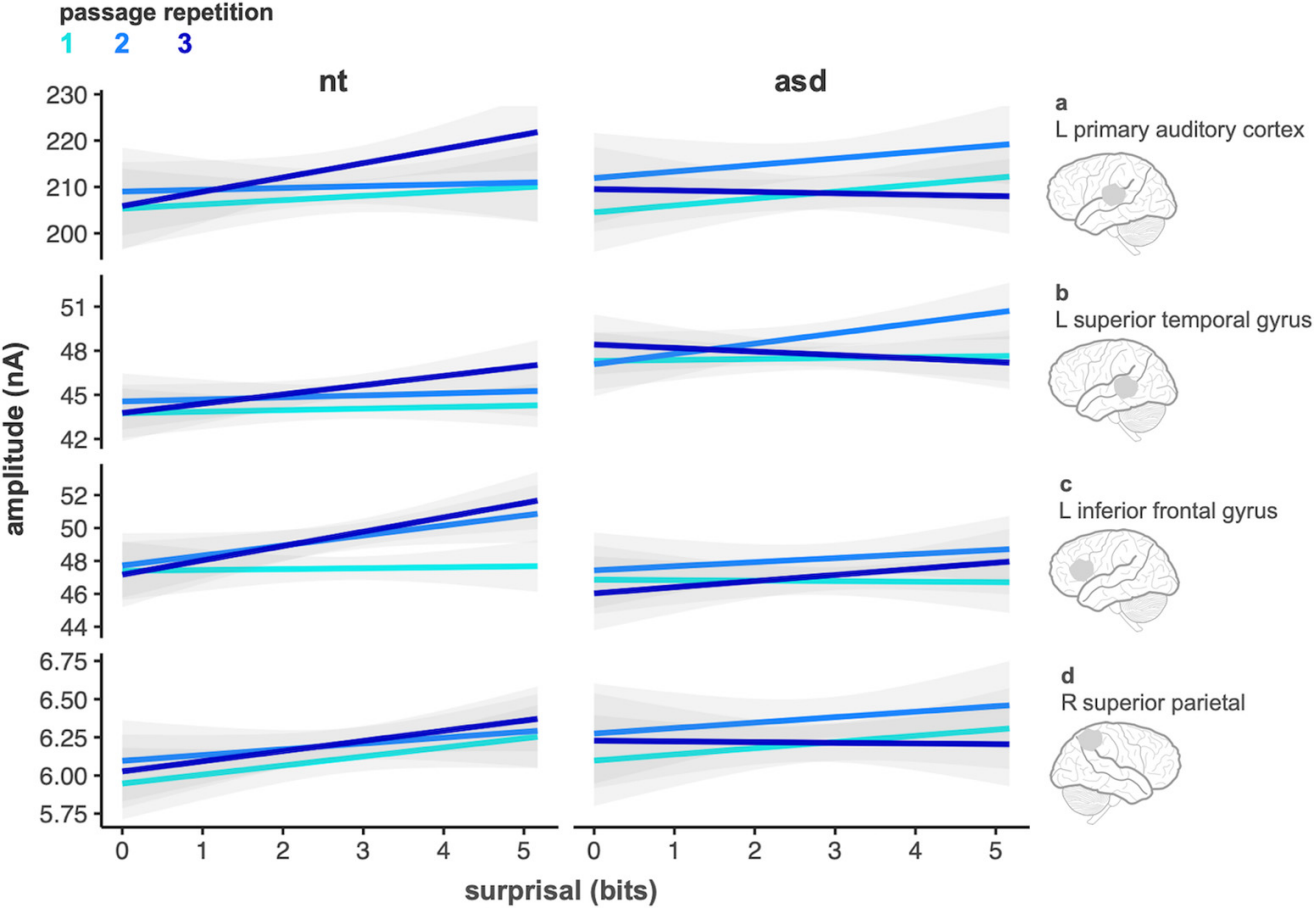
Linear effect of evoked response amplitude (averaged across time windows) as a function of syllable surprisal for each group and ROI across the first, second, and third passage repetitions (light blue to dark blue lines). Gray shading represents SE.

Figure 4 shows the linear effect of evoked response amplitude as a function of syllable surprisal for each group, ROI, and passage repetition. These plots are averaged across time windows for ease of visualization (the statistical results, summarized below, showed no higher-order interactions with time). ANOVA results are reported in Table 2.

**Table 2:** Results of an ANOVA comparing mean amplitude across group (ASD and NT), syllable surprisal, passage repetitions, ROIs, and time windows

Main effects	df, residual	*F*	*p*
Surprisal	1, 137925	21.89	0.000
Time window	2	1.21	0.298
Repetition	2	11.44	0.000
ROI	3	64184	0.000
Group	1, 27	0.01	0.921
Two-way interaction			
Surprisal × time window	2	0.19	0.823
Surprisal × repetition	2	0.70	0.494
Time window × repetition	4	0.13	0.971
Surprisal × ROI	3	9.82	0.000
Time window × ROI	6	0.47	0.827
Repetition × ROI	6	4.32	0.001
Surprisal × group	1	4.59	0.032
Time window × group	2	0.03	0.966
Repetition × group	2	1.81	0.164
ROI × group	3	6.95	0.001
Three-way interaction			
Surprisal × time window × repetition	4	0.13	0.972
Surprisal × time window × ROI	6	0.06	0.999
Surprisal × repetition × ROI	6	0.85	0.531
Time window × repetition × ROI	12	0.07	0.999
Surprisal × time window × group	2	0.29	0.752
Surprisal × repetition × group	2	3.09	0.046
Time window × repetition × group	4	0.22	0.926
Surprisal × ROI × group	3	0.68	0.566
Time window × ROI × group	6	0.02	0.999
Repetition × ROI × group	6	0.44	0.853
Four-way interaction			
Surprisal × time window × repetition × ROI	12	0.09	0.999
Surprisal × time window × repetition × group	4	0.17	0.955
Surprisal × time window × ROI × group	6	0.13	0.992
Surprisal × repetition × ROI × group	6	4.32	0.001
Time window × repetition × ROI × group	12	0.04	0.999
Five-way interaction			
Surprisal × time window × repetition × ROI × group	12	0.11	0.999

A neural index of learning would be reflected by an increase in the amplitude of the evoked response as a function of surprisal and passage repetition. We tested whether this interaction effect differed across groups, ROIs, and time windows. We found a key four-way interaction showing surprisal by passage repetition varied by group and ROI (*p *=* *0.001, η_p_^2^ = 0.95). This interaction reflects the fact that a positive slope for the effect of surprisal emerged in the third repetition for NT participants but not for ASD participants. The pattern of positive slope in the third repetition in the NT group is consistent across the left LAC, pSTG, and IFG regions and differs for the right parietal region.

We further break-down this interaction effect. In LAC (Fig. 4*A*), for the NT group, the effect of evoked response amplitude across surprisal (slope of blue lines) shows a positive incline in the third passage repetition relative to the first two passage repetitions (β = 4.53, CI_95%_ = [2.85, 6.21]). This pattern of data differs for the ASD group where we observe a flat trend in passage repetition three in the LAC (β = −1.66, CI_95%_ = [−2.86, −0.44]), relative to the first two passage repetitions. In LSTG (Fig. 4*B*), for the NT group, the blue line is overall flat for the first two repetitions and shows a positive trend in the third passage repetition. Meanwhile, the ASD group’s blue lines reflect a slight negative trend in the first and third repetitions and a positive trend in the second repetition. In the LIFG (Fig. 4*C*), we again observe overall flat blue line for the NT group in passage repetition one and a positive trend in the second and third repetitions; no such pattern is observed for ASD across all three repetitions. In the right superior parietal, as expected, we observe no learning response across passage repetitions in both NT and ASD groups (Fig. 4*D*).

The ANOVA showed a marginally significant three-way interaction of surprisal by repetition by group effect (*p *=* *0.046, η_p_^2^ = 0.82). Additionally, we observed several significant two-way interactions: the effect of surprisal varied across ROI (*p *<* *0.001, η_p_^2^ = 0.96), brain activity across the three repetitions varied by ROIs (*p *=* *0.001, η_p_^2^ = 0.95), the effect of surprisal varied by group (*p *=* *0.032, η_p_^2^ = 0.77), and brain activity at the three ROIs varied by group (*p *=* *0.001, η_p_^2^ = 0.94). We also observed several lower-order significant effects including main effects of surprisal (η_p_^2^ = 0.94), passage repetition (η_p_^2^ = 0.94) and ROIs (η_p_^2^ = 1.0; all *p *<* *0.001). The main effect of time window (η_p_^2^ = 0.64) and group (η_p_^2^ = 0.01) were not significant. The five-way interaction between surprisal, time window, repetition, ROIs, and group was not significant.

## Discussion

The present study used surprisal to investigate the neural mechanisms underlying speech segmentation in typical development and in children with ASD. Speech segmentation, foundational to language acquisition, requires the integration of top-down and bottom-up cognitive processes. To this end, we proposed two possible hypotheses as to why children with ASD might struggle to use distributional cues to find words in speech: a sensory-differences hypothesis that suggests potential deficits in the bottom-up early sensory processing of auditory input, and a prediction-differences hypothesis related to potential deficits in the high-order analysis of concatenated input. To investigate these two hypotheses, we used MEG to examine the functionality of the left primary auditory cortex, left posterior STG, and left IFG region during a passive language listening paradigm. Our key interest was a neural index of learning, measured as an increase in the amplitude of the evoked response as a function of surprisal. We expected this interaction to emerge with repeated exposure to the language paradigm. Critically, we tested whether neural responses differed across groups and ROIs. We observed the neural index of learning in typically developing children, but not in the children with ASD, across all three ROIs. These data speak to two competing hypotheses.

First, prior literature on speech and sound processing have shown that children with ASD present with low-level auditory processing deficits, such as disruptions or delays in early neural responses to both verbal and non-verbal acoustic stimuli (Bomba and Pang, 2004; Jeste and Nelson, 2009; Edgar et al., 2014, 2015). In fact, the set of children with ASD in this sample previously showed atypical responses to phototactically illegal, in comparison to legal, sequences (Brennan et al., 2016b). Our LAC and pSTG results are consistent with the sensory-differences hypothesis that suggests a possible disruption in initial acoustic processing may have led to difficulties in extracting speech sound patterns from natural fluent speech (Roberts et al., 2010, 2011).

Second, research into the development of auditory pathways in ASD show atypical development of white matter and cortical function within the auditory and language systems (Berman et al., 2016), such as delayed STG auditory 100-ms responses (Roberts et al., 2010) and atypical hemispheric lateralization of auditory responses (Stroganova et al., 2013). These patterns of responses in auditory processing may be because of the documented deficits of orienting attention (Whitehouse and Bishop, 2008). ASD children in this study showed a varied pattern of neural responses to syllable sequences as compared with neurotypical peers, within and across all three ROIs. Specifically, the IFG results are in line with the prediction-differences hypothesis. Prior work has suggested that the language network’s feed-forward mechanisms of higher-order computations might be particularly impaired in those with ASD and poor language learning outcomes (Courchesne and Pierce, 2005; Redcay et al., 2008). While speculative, such impairments have the potential to propagate extraction and integration learning deficits in ASD, especially in the beginning phases of learning.

Behavioral measures of statistical learning suggest that ASD children could be as sensitive to statistical regularities as their typically developing peers (Haebig et al., 2017), across paradigms with (Scott-van Zeeland et al., 2010) and without (Mayo and Eigsti, 2012) additional cues to segmentation. In the present study, most of the ASD children were unable to identify the target syllable pairs heard within the novel fluent speech relative to a foil. Performance for this group of children with ASD was significantly below chance, suggesting that some learning may be happening within the 6-min exposure. The pattern of data suggests that children with ASD were able to recognize some syllable components that were part of words used in the postscan behavioral test, but not the syllable sequences that formed the target words. One interpretation of these findings is consistent with to our second hypothesis relating to higher-order analysis of linguistic events. Children with ASD may have been sensitive to the frequency of syllables presented but failed in the appropriate grouping of syllable sequences given the distributional cues. This is an interesting finding that warrants further investigation.

Our NT and ASD children did not differ in their phonological competence, although they differed on measures of attention, syntax, and pragmatics. Children with ASD showed normative performance on the phonological awareness tasks that ask children to segment and manipulate word sounds (e.g., elision, CTOPP), but poorer performance on syntax tasks (e.g., formulating sentences, CELF-4) that tap into children’s knowledge of language structure. Observed differences in neural learning patterns within left hemisphere regions and poor statistical learning performance in ASD may be revealing of ASD children’s underlying difficulty in extracting linguistic structure or sequence learning that extends beyond processing of single speech sounds. However, exploratory bivariate correlations between language and attention measures with experimental task performance indicated no meaningful trends (*r *=* *0.01–0.37). The sample size significantly limits our ability to examine the links between the current paradigm and children’s language or cognitive skills. In future work, we aim to take a closer look at defining subpopulations of children with ASD and their learning outcomes.

The Italian statistical learning paradigm, adapted from Hay et al. (2011), maintained virtually all complexities found in natural speech with the exception that the transitional probabilities between syllable sequences were precisely manipulated in a subset of words. By specifically examining prediction-based processing demands with the measure of surprisal, we were able to assess the computational nature of statistical learning across a range of unexpectedness values. This allowed us to control and test for statistical learning effects of relatively moderate and highly predictive syllable sequences within a continuous and varied range of syllable probabilities. Prediction has been implicated as an important component of early learning (Romberg and Saffran, 2013), and some suggest prediction plays a major role in the underlying impairments observed in ASD (Sinha et al., 2014). This hypothesis suggests that tracking of statistical regularities in ASD might compare to neurotypical peers when the environment is relatively stable, and perhaps with longer exposure time (e.g., 21 min in Mayo and Eigsti, 2012). However, when tasks involve varying distribution of events (e.g., range of probabilities), integration of new events with prior experiences may be more difficult for children with ASD, resulting in learning differences between the two groups.

The use of a naturalistic language paradigm, combined with MEG imaging, is one of the key innovations of this study. Previous studies of speech segmentation that vary the type and number of speech cues available to learners have found differences in the neural activity across manipulations, despite participants’ inability to behaviorally detect differences between conditions. This has been documented in a sample with typically developing children (McNealy et al., 2010; Scott-van Zeeland et al., 2010) and adults (McNealy et al., 2006) using fMRI. Scott-van Zeeland et al. (2010) found that both children with and without ASD were at chance in their behavioral learning performance. Importantly, they differed in their neural responses. First, the authors found that patterns of brain activity in the fronto-temporo-parietal network changed with the increase in the number of cues to word boundaries, but only in the group of typically developing children. Second, the authors observed a lack of frontal lobe engagement during task of speech processing in children with ASD. Lastly, children with more severe communicative deficits showed fewer changes in brain activity with increased exposure to speech. Our results parallel these findings and provide corroborating support for the hypotheses that integration of top-down and bottom-up cognitive processes are involved in successful speech segmentation, which may be impaired in children with ASD. In the present study, we found no evidence of a timing effect in relation to early speech processing in the auditory cortex and later analysis in higher-level auditory and speech processing regions. This an interesting null result that warrants further investigation with a more granular experimental design.

The use of the beamforming method for localization introduces some limitations, such as possible differences in the quality of fit between ASD and NT groups. Thus, we cannot rule out an anatomic-based explanation of our results. However, we have two reasons to think such an explanation is not likely. First, potential anatomic differences in ASD and NT may be smaller than the spatial specificity of the beamformer. Second, the anatomical differences in the left hemisphere between ASD and NT groups pointed out by Berman et al. (2016) emerge at later ages than the 8- to 12-year-old range studied in our sample. To test this reasoning in future studies, we could measure the statistical fit of the beamforming method across the two groups or acquire individual MRI anatomical scans for each participant to estimate source localizations with more precision.

In sum, the present study offers novel evidence investigating the neural mechanisms underlying statistical learning using a naturalistic language paradigm, in typical development and in children with ASD. Results show neural and behavioral effects of speech segmentation specific to syllable-level surprisal, extending previous work by examining statistical learning from two perspectives – input-driven auditory processing and higher-order predictive processing. These findings offer insight into the cognitive mechanisms foundational for language acquisition and helps inform our understanding of development across different populations of learners.

## Synthesis

Reviewing Editor: Alfonso Araque, University of Minnesota Decisions are customarily a result of the Reviewing Editor and the peer reviewers coming together and discussing their recommendations until a consensus is reached. When revisions are invited, a fact-based synthesis statement explaining their decision and outlining what is needed to prepare a revision will be listed below. The following reviewer(s) agreed to reveal their identity: NONE.

The reviewers have found merit and interest on the findings reported. I concur with the reviewers' comments expressing the interest of the study. However, they also expressed some concerns about several issues that need to be addressed and clarified. I also consider pertinent the concerns expressed. We believe that addressing the concerns is necessarily required to firmly validate the results and support the conclusions. We also believe that those concerns can be addressed in a relatively short time, requiring less than 2 months' work.

I am looking forward to receiving a revised version of the manuscript.

Specific comments of the reviewers:

Reviewer 1.

The study at hand describes a study investigating statistical learning in natural speech and its neural correlates (i.e., magnetencephalography, MEG) in children with Autism Spectrum Disorder (ASD) and their typically developing peers. Neural responses to continuous speech were recorded and three regions of interest, namely the left primary auditory cortex, the left posterior superior temporal gyrus (STG), and the left inferior frontal gyrus (IFG) were analyzed. Further, the authors varied the probability of how syllables are likely to co-occur together. The results showed that typically developing children showed specific neural responses in all three regions, but children with ASD did not. Specifically, typically developing children showed an increase in their neural response in the last of three runs (i.e., effect of learning) especially when syllables were less likely to co-occur and therefore, surprisal was high.

While this study is generally interesting, I had some difficulties while reading the manuscript. Next to others, these difficulties were related to the motivation of the study, to the methods/analyses strategy, and to the discussion of the study.

In the following, I will describe my concerns in detail along with some more minor comments and I strongly suggest revising the manuscript, before it can be accepted for publication.

Major comments:

1. Study motivation:

The exact motivation for investigating statistical learning by using natural speech in children with ASD does not become clear. Why is it important to investigate this and what would the reader learn from this research question? This should be described specifically to enhance comprehension of the study. I suggest going through the whole manuscript, especially the introduction, and rephrasing the corresponding paragraphs to make be more specific. In addition, the following comments are also related to the motivation and aim of the study and hopefully help the authors to change the introduction to enhance comprehension and to convey the aim and motivation of the study.

- Page 3, line 17 and following: Here, the authors state the aim of the study. However, when they want to state the aim this early in the manuscript, they should also motivate the study, why it is important to investigate this research question?

- Page 4, line 34: Does this mean that the authors, even though artificial language learning was intact in ASD, think that it should be tested in natural speech, where individuals with ASD were shown to have deficits? If yes, why not explicitly state this?

- Page 4, line 35 and following: The authors state that attention, extracting global regularities, etc. were shown to be deficient in ASD. It should be described how these abilities are relevant for statistical learning in natural speech.

Further, the authors state that the deficient abilities (i.e., attention, etc.) are relevant for statistical learning and by this, they indirectly say that statistical learning is probably deficient in ASD. However, before the authors describe that statistical learning was found to be typical in ASD, at least on the behavioral level and when tested artificially. This is a bit confusing and needs to be clarified. As well a better motivation why this needs to be investigated is needed.

- Page 4, line 45: Why should children with ASD have difficulties in extracting statistical regularities from fluent speech, but not from other material? Or do I not understand the whole introduction? This needs to be clarified.

- Page 5, line 59: What is the connection of the following statement to the research question. The authors should specifically clarify this. “...surprisal is thought to indicate the cognitive effort required to integrate a word within its current context...”.

- Page 5, line 61: Why was the focus of the study to investigate statistical learning in continuous speech in children with and without ASD ? Why should this be done and what would be the additional value of the study? The authors should clarify.

2. Analyses:

1) the authors look at three regions of interest that are related to language processing, namely the left auditory cortex, the left posterior STG, and the left IFG. However, I was wondering whether the found effect is specific to the defined regions of interest or whether it is a more general effect. Therefore, a control analyses is needed with either regions that are not directly associated with language processing, such as V1, etc., or with the right contralateral homologous regions; and I strongly suggest following the suggestion of control analyses to enhance the strength of the manuscript. Otherwise, it could also be the case that the effects found are more general due to differences in statistical learning, independent of language/speech processing.

2) The question arises how the findings are related to language abilities. On page 16, line 313, the authors state that “most ASD children were unable to identify the target syllable pairs...”, which could be related to general language difficulties and the reader might ask the question whether this was expected or whether this was controlled for. Even though the sample size is small, the authors should post-hoc test for associations with language abilities or they should control for them. This will help them to strengthen the discussion and interpretation.

The following comments concerning the discussion are further related to this comment.

3. Discussion:

1) The authors make a strong claim of sensory deficits in ASD to cascade to difficulties in extracting speech sound patterns from natural fluent speech (page 16, line 317). However, the authors did not test the effect of sensory deficits directly and should therefore be careful with their conclusion.

2) page 17, line 332 and following: The authors state that prior studies using artificial languages with reduced phonological and structural complexity did not observe any deficits in ASD. Here, the first thing that comes into my mind is that language abilities have an effect on the results or are directly influencing the results, which should be tested. Importantly groups differ in their language abilities, further stressing the fact that language abilities might impact the results dramatically. If this was expected, the authors need to state this explicitly and they need to analyze this directly.

3) page 17, line 339 - 340: “...integration of new events with prior experiences may be more difficult for children with ASD, resulting in learning differences between groups.” But maybe this is specific to language abilities? This should be clarified.

More minor comments:

Abstract:

- “word discovery” is an unusual expression and should be changed.

- The “sensitivity to the regularities within the linguistic stream” is not only relevant for vocabulary, but also for grammar, which should be mentioned.

- What is the “information processing metric of surprisal”? This might not be entirely clear to the reader and I suggest explaining it.

- The authors should introduce the abbreviations used in the abstract, namely MEG, IFG, and STG

- What is syllable-level surprisal? This needs to be explained, otherwise it is difficult to follow the abstract.

- “No such pattern was observed for ASD” - the pattern did not become entirely clear.

- “... speech segmentation and processing.” So far, it is unclear how speech segmentation was tested, was it?

- It also does not become clear from the abstract how “predictive processing in statistical learning...” was tested.

Significance Statement:

- Page 2, line 4: A sensory deficit was not really investigated in the present study, right?

- Page 2, line 7: It is still not clear what “syllable-level surprisal” is

- Page 2, line 9: How is “... examining the computational nature of speech segmentation...” informing developmental and cognitive theories of language acquisition? This does not become clear so far.

Introduction:

- Page 3, line 25 and following: The authors describe a study by Scott-van Zeeland and colleagues (2010) and describe that they found “... fMRI habituation effects were found in fronto-temporal-parietal networks in typically developing children, but not in ASD children... “. What do these results mean? How did van Zeeland and colleagues interpret the results? What might be the reason for the differences between neural and behavioral responses - This should be clarified and described in more detail.

- page 4, line 51: The concept of surprisal needs to be explained in much more detail before.

- page 5, line 62 and following: This paragraph is difficult to follow and should be rephrased. The key steps of speech processing should be explained in more detail.

- page 5, line 67: What is “a neural index of learning”? What “effect in the amplitude", how would this effect look like?

- page 5, line 69: The interaction effect needs to be specifically explained, otherwise it is difficult to understand what the authors mean.

Methods:

- Page 6, line 81 to 82 & line 86 to 87: It should at least be mentioned what the questionnaires aim to test.

- page 6, line 93 and following: What is the ADOS. This should be explained.

Generally, the diagnostic instruments and questionnaires need to be explained in much more detail. What are they for? Are they standardized? What are the cutoff criteria and how were they defined?

- page 6, line 95: How was the intellectual cutoff defined and who suggested the cutoff? Basically, I am asking what the cutoff was based on, because it seems quite low.

- page 7, line 110: Why did the ASD participant not complete the experimental task? This should be explained in more detail.

- page 8: The behavioral measures have not been mentioned so far. Therefore, the question arises why they were measured? Especially because the authors did not use them for diagnosis, nor for analyses. The behavioral measures should be motivated already in the introduction.

- page 8, line 147: phonological awareness should be shortly defined.

- page 9, line 154: Why were attention and executive functions measured?

- page 10, line 174 and following: The authors state that the experiment was part of a longer MEG session. Was statistical learning tested directly after the continuous speech experiment in the MEG or after the longer MEG session? This needs to be clarified.

- page 10, line 183: I fear tat the individual adjustment of volume possibly impacts the results?

- page 11, line 213 and following: The selection of ROIs was based on previous findings in adults. Were the findings generally related to statistical learning or to statistical learning in the speech domain? This needs to be clarified.

- page 12, line 240: Why did ASD children not complete the post-scan?

Discussion:

- page 15, line 300 and following: The motivation for the present study is still unclear. Why should deficits in extracting regularities from natural fluent speech in ASD be investigated?

- page 15, line 305: What would the neural index of learning be?

- page 15, line 306: Please explain what the “pattern of learning would look like”. The authors should be more specific.

- page 15: The footnote has no correspondence in the main text. At least I did not find it.

- page 17 to page 18; line 353 to 354: How are orienting attention deficits explaining the patterns of responses in auditory processing? This should be explained in more detail.

Reviewer 2.

The manuscript “Predictive processing during a naturalistic statistical learning task in ASD” shows interesting findings in differences in statistical learning in ASD relative to their TD peers, and particularly interesting findings in neural MEG data. While the manuscript is well written and clear, my main concern is that there is a huge missed opportunity with the current analysis. The primary reasoning for studying statistical learning in ASD is its potential impact on language and social communication. While the authors recorded data on these individual attributes (and attention), there is no connection made between the statistical learning data and these measures. While the N is relatively small for such an analysis, a non-parametric analysis, explicitly noting the small N, would make this paper much more meaningful, even if only effect sizes are reported. This is the primary goal of this entire area of research, and it’s importance should not be overlooked. Specifics and other comments are below.

Page 3-"typically developing” has NT as an acronym. Is “neurotypical” what is meant here? The abbreviation is mismatching if not. Also, once NT is introduced the first time, use it throughout.

Page 4 - spell out MEG in its first use. It’s defined later on in the paragraph instead of in the first instance.

Page 4 - cite missing for “and show atypical sound and speech processing”.

Page 4 - It would be useful to define surprisal.

Page 4 - “have a delayed mismatch field response” - what does this mean functionally?

Page 5 - No ASD vs NT hypotheses?

Page 6 - was the administer of the ADOS clinical or research reliable?

Page 6 - FSIQ is used but not identified in the first instance of use.

Page 6 - “FSIQ was within standard error of the suggested cutoff for intellectual disability” - define this, as it varies across settings.

Page 6 - If two participants that were included were below the inclusion cutoffs, then these really aren’t inclusion criteria. It would be simpler just to describe what the actual ranges were.

Page 7 - “Exclusion criteria for initial NTs ... included any history of developmental delay, learning disability, head injury with loss of consciousness, neurological illness, environmental deprivation, or psychopathology, or a first-degree relative with ASD.” - aside from the first-degree relative criteria, were theses other criteria also applied to the ASD group?

Page 7 - “Participants listened passively to approximately six minutes of a naturally produced” - Figure 1 says 7 mins. Which is correct?

Table 1 - please include actual p-values and effect sizes. And please include effect sizes through the text for each statistical result.

Page 10 - “To keep attention and restrict head or body movements, we asked the children to look at a ceiling poster above the MEG bed that depicted colorful fish swimming in a coral reef.” This phrasing makes it sound like this setup increases attention to the stimuli. Please rephrase.

Page 11 - “approximately15-20 cm” Space missing

Page 12 “The root-mean-square (RMS) time-course within three consecutive 100 ms time-bins (Teinonen et al., 2009): 200-300 ms, 250-350 ms, and 300-400 ms” - there’s something off here, these aren’t 3 100ms consecutive time bins. If these are correct, they are overlapping and thus non-independent, which will impact statistical testing.

Results - report effect sizes throughout.

Page 13 - For the results starting on ln 259, these results are different from Scott-Van Zeeland (2010) who found no evidence of above chance performance in both ASD and TD children using an auditory SL task and chalked it up to using young children in their study (they didn’t expect to find evidence of learning). Perhaps provide some clarity as to why these findings were different?

For the neural indices of surprisal, would one not have predicted that with each subsequent repetition would have been higher than the last, as oppose to a slope within each repetition? I’m wondering if it may be more advisable to plot these as a single slope across the entire experiment as opposed to three separate lines.

For the following paragraph, were these descriptions of slopes run through statistical tests on their own? Acknowledging the overall interaction in the ANOVA is one thing, but to make these claims of meaningful slopes, these should also be run independently.” In LAC (Figure 4a), for the NT group, the effect of evoked response amplitude across surprisal (slope of blue lines) is relatively flat for the first two passage repetitions and then shows a positive incline in the third passage repetition. This pattern of data differs for the ASD group where we observe a clear negative trend across all three passage repetitions in this ROI. In LSTG (Figure 4b), for the NT group, the blue line is overall flat for the first two repetitions and shows a positive trend in the third passage repetition. Meanwhile, the ASD group’s blue lines reflect a slight negative trend in the first and third repetitions and a slight positive trend in the second repetition. Lastly, in LIFG (Figure 4c), we again observe overall flat blue lines for the NT group in passage repetition one and two with a clear positive slope in the third repetition. No such pattern is observed for ASD across all three repetitions.”

Behavioral results - Given the significantly below chance performance of the autistic children, it does seem that they are in fact responding to the statistics of the language, they’re just responding in the wrong direction. (otherwise they would be at chance). What do you make of this?

Also, in the related Figure 3, please including in the caption what the error bars represent

There is a broad literature discussing sensory irregularities in ASD - can you rule out that differences may be caused by lower-level sensory disruptions?

In this paradigm, language processing was pleasured using a number of instruments, CTOPP, CELF, and TOPS. Given that the main thrust of the reasoning behind investigating SL is on it’s relationship to language, it seems like a very significant missed opportunity to not test whether these deviances in typical SL relate to the actual measures of language. This would be an extremely easy analysis and would greatly enhance the impact of the manuscript, even if it were preliminary/exploratory.

The footnote at the bottom of page 14/15 is split.

Discussion and general -tone down the description of novelty for using naturalistic language. Studies have been conducted that include stressed language & prosodic cues.

Discussion: “"This hypothesis was not supported by our results; most of the ASD children were unable to identify the target syllable pairs heard within the novel fluent speech relative to a foil. This learning deficit is consistent with a second hypothesis based on evidence for atypical neural responses to speech sounds in temporal regions in ASD (e.g., Roberts et al., 2011; Roberts et al., 2010), which predicts that sensory deficits could cascade to difficulties in extracting speech sound patterns from natural fluent speech.”- this needs to be more nuanced. It would be one thing if autistic children were at chance, but they were significantly below chance, which means there is some learning going on. It would be beneficial to discuss how this may arise and what it may mean.

Differences were seen in all ROIs, with no impact of time. A cascade effect would predict that the divergence in early auditory cortex should be earlier than in higher-level auditory and speech processing regions. It would be beneficial for the reader to discuss what the lack of timing * ROI interaction indicates.

Citation needed: “Our results are consistent with a cascade effect hypothesis that suggests sensory deficits in ASD may have led to difficulties in extracting speech sound patterns from natural fluent speech.”

"These group differences are consistent with evidence that early neural responses to acoustic stimuli are disrupted in ASD (Bomba & Pang, 2004; Jeste & Nelson, 2009) and may impact speech segmentation and processing.” - you have the data to test this, and you should.

"These patterns of responses in auditory processing may be due to the documented deficits of orienting attention (Whitehouse & Bishop, 2008).” - Measures of attention were collected as well - this is testable with the current data set.

"However, the sample size limits our ability to examine the links between the current paradigm and children’s language skills.” Referring to the above two comments, despite the smaller N, non-parametric tests can still be conducted, and at the very least effect sizes reported. Again, this would make the paper much more meaningful.
